# The Bottleneck Effect: Wait Times for Adult ADHD Assessment at a Private Clinic in Australia

**DOI:** 10.1192/j.eurpsy.2025.1610

**Published:** 2025-08-26

**Authors:** R. D. Mendonsa, H. T. Jayasooriya

**Affiliations:** 1 Mental Health and Wellbeing, Western Health, Melbourne, Australia

## Abstract

**Introduction:**

Public sector mental health services in Australia typically do not provide Adult ADHD assessment or treatment, creating a significant reliance on private sector care. Consequently, the demand for private ADHD services has surged, resulting in extended wait times for assessment and treatment.

**Objectives:**

This study aimed primarily to evaluate the wait times for Adult ADHD assessments for patients referred by GPs to a private clinic. A secondary aim was to analyze the relationship between sociodemographic and clinical variables, including illness characteristics and timing of diagnosis.

**Methods:**

Data were collected through retrospective file reviews of consecutive patients referred to the authors’ private clinics for Adult ADHD assessment between January 2023 and October 2024. Patients included in the study met the criteria of an eventual clinical diagnosis of Adult ADHD. Data collected included sociodemographic details, ADHD subtype, psychiatric comorbidities, and wait times for initial psychiatric consultations. Total sample was 68.

**Results:**

Wait times ranged from 10 days to 305 days, with a mean wait time of approximately 4 months (112 days). Almost 30 % of the patients referred had wait time of more than 4 months. The sample comprised nearly equal numbers of male and female patients (33 vs. 35), with ages ranging from 17 to 56 years (mean age: 28.35 years). The majority (68%) were diagnosed with Adult ADHD - Combined Presentation, while 32% had the Predominantly Inattentive Presentation. Nearly all patients received their ADHD diagnosis in adulthood, with less than 5% having a childhood ADHD diagnosis.

**Conclusions:**

There are significant delays in acessing appropriate care for people with Adult ADHD in Australia. Improvement in mental health policy and service delivery with regard to ADHD services is essential if this barrier to access appropriate care has to be overcome.

**Disclosure of Interest:**

None Declared

